# Mesenteric Vein Thrombosis Following Platelet Transfusion in a Patient with Hemorrhagic Fever with Renal Syndrome: A Case Report

**DOI:** 10.1055/s-0038-1669456

**Published:** 2018-08-27

**Authors:** Anne-Marie Connolly-Andersen, Johan Rasmuson, Mikael Öman, Clas Ahlm

**Affiliations:** 1 Department of Clinical Microbiology, Infectious Diseases, Umeå University, Umeå, Sweden; 2 Department of Surgical and Perioperative Sciences, Surgery, Umeå University, Umeå, Sweden

## Introduction

Viral hemorrhagic fevers (VHFs) are typically characterized by life-threatening thrombocytopenia and tendency to bleed. Still, there are gaps of knowledge regarding risks and benefits of platelet transfusion for patients with severe thrombocytopenia during VHF and other infectious diseases. 1 2 3 4 Hemorrhagic fever with renal syndrome (HFRS) is a mild rodent-borne VHF, caused by Puumala virus, endemic in central and northern Europe. The infection is characterized by fever, nausea, back- and headache, thrombocytopenia, and transient renal impairment. We have shown that HFRS is a risk factor for both arterial and venous thrombosis. 5 6 We here report a case of a patient with HFRS with prolonged thrombocytopenia who developed thrombosis shortly after receiving platelet transfusion.

## Case Report

**Fig. 1:**
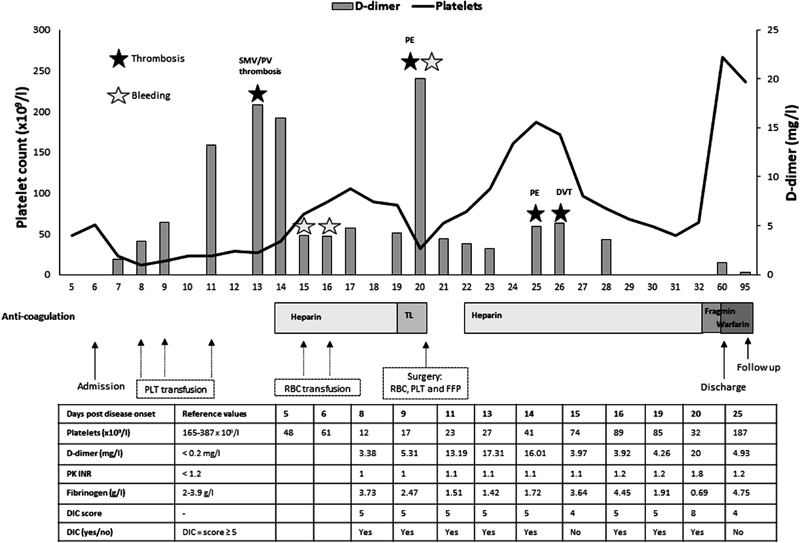
A case with hemorrhagic fever with renal syndrome and mesenteric vein thrombosis in relation to interventions and coagulation disturbances (the time line is presented as days post disease onset on the *x* -axis). The levels of platelets and D-dimer are presented on the *y* -axes and other markers for coagulation disturbances in the table. Episodes of thrombosis and bleeding are illustrated by colored stars (black for thrombosis and red for bleeding). The different interventions are indicated below the time line. DIC score is calculated according to the International Society of Thrombosis and Haemostasis guidelines as previously described. 8 DIC, disseminated intravascular coagulation; DVT, deep vein thrombosis; FFP, fresh frozen plasma; PE, pulmonary emboli; PLT, platelet; PV, portal vein; RBC, red blood cell; TL, thrombolysis; SMV, superior mesenteric vein.

A 73-year-old woman with no previous comorbidities or family history of hematological disorders or hypercoagulability was admitted to the Infectious Disease Clinic due to suspected HFRS and dehydration. Two weeks prior to disease onset, she had been exposed to bank voles while cleaning out a cabin. For 6 days following disease onset, she had been ill with fever, chills, weakness, low urine production, and difficulties eating and drinking. The patient had positive serology for Puumala virus thereby confirming the HFRS diagnosis. Laboratory tests taken the day before admission revealed thrombocytopenia (platelet count: 48 × 10 ^9^ /L), impaired renal function (creatinine: 278 μmol/L), and leucocytosis (white blood cell count: 14 × 10 ^9^ /L). Upon admission to the hospital, the platelet count had increased to 61 × 10 ^9^ /L and creatinine increased to 370 μmol/L indicating clinical progression to the oliguric stage of HFRS. However, the platelet levels decreased to 12 × 10 ^9^ /L on days 8 to 9. The treating physicians decided to transfuse platelets on days 8, 9, and 10 due to the high risk of spontaneous bleeding. Despite transfusion with three platelet units, the patient remained severely thrombocytopenic with platelet counts below 50 × 10 ^9^ /L during days 8 to 13. The case is summarized in Fig. 1 . Criteria for disseminated intravascular coagulation (DIC) were fulfilled from day 8 (see Table 1 for an overview of criteria). 7 8 On day 13 (2 days after the last platelet transfusion and a platelet count of 27 × 10 ^9^ /L), the patient falls ill with abdominal pain which increases in severity during the evening. An abdominal computed tomography (CT) shows congestion and ischemia in the terminal ileum due to a thrombus in the superior mesenteric vein (SMV) reaching up to the portal vein (PV). The hematologist advised against thrombolysis due to thrombocytopenia in combination with a known mild VHF, which could increase the risk of bleeding. A national coagulation expert is consulted for further advice, who recommends anticoagulant treatment with heparin in a “careful” dose. Heparin at a dose two-thirds the national recommended dose is initiated with the aim of APTT 1.5 times the baseline value (40–50 s). The patient therefore receives a bolus dose of 4,000 units heparin followed by transfusion of 24,000 units heparin per day. The following day (15), the patient has gastrointestinal bleeding and a decrease in hemoglobin values from 100 to 89 g/L. According to the surgeon consultant, the stasis caused by the SMV and PV thrombus damages the intestinal mucosa leading to the observed gastrointestinal bleeding. Heparin is therefore continued in the same careful dose and the patient receives one unit red blood cells (RBCs) that day and the following day (16). The following criteria had to be fulfilled before mesenteric phlebography and thrombolysis via catheter could be considered: (1) platelet levels greater than 100 × 10 ^9^ /L, (2) no bleeding, and (3) the patient can tolerate a full-dose heparin. On day 19, the patient fulfilled these criteria and underwent mesenteric phlebography via interventional radiology. Using the percutaneous transhepatic route to the PV, a hydrolyser 7F, double lumen, over-the-wire thrombolysis (Hydrolysis, Cordis Europe NV, Roden, the Netherlands) was used to perform a mechanical thrombolysis of the PV followed by pharmacological thrombolysis with tPA (Actilyse infusion: 0.8 mg/hour). A notation from the surgery department states that the previous heparin treatment aiming for 1.5 times APTT had been unsuccessful in decreasing the size of the SMV and PV thrombus. A control angiography 6 hours postsurgery shows that the thrombus distally in the SMV has been removed. There is still a thrombus between the portal and the splenic vein; therefore, the catheter is moved further into the area of thrombosis and thrombolysis by Actilyse administration is continued. At this time, there is no contrast leakage as a sign of hepatic bleeding. The patient stays in the intensive care unit (ICU) with local hydrolysis via the catheter. The following day (20), the patient becomes hypotensive with systolic blood pressure down to 75, and has signs of peritonitis. The levels of the fibrin degradation product D-dimer increases to 20 mg/L and fibrinogen decreases to 0.69 g/L. A CT thorax/abdominal scan shows an ongoing expanding hepatic intraparenchymal arterial bleeding. In addition, the CT scan shows presence of pulmonary emboli. Since the patient has a propensity for bleeding and thromboembolism, arterial intervention via the femoral artery into the aorta and then out into the common hepatic artery with coiling was not an option. Instead an emergency surgery procedure is performed with ligation of the right hepatic artery in the hepatoduodenal ligament, which stops the bleeding. In addition, the anticoagulant therapy is discontinued that day and the patient is tended in the ICU in a respirator. On day 21, the anticoagulant therapy is readministered at a low dose. The patient is extubated on day 22 and the anticoagulant therapy is increased to a target of APTT 60s due to remaining portal thrombi and peripheral pulmonary emboli. On the evening of day 25, the patient develops acute dyspnea, and oxygen saturation decreases to 88% with 4 L of oxygen and tachycardia. A CT pulmonary angiography shows pulmonary emboli in the right and left pulmonary arteries and peripherally in the pulmonary lobe arteries. The patient is transferred that evening to the ICU with heparin treatment at a target of APTT 85s. An ultrasound of the peripheral extremities (day 26) shows bilateral deep vein thrombosis in the posterior tibial veins. Since the APTT remains difficult to adjust (ranging from over 180s to the therapeutic target of 80s) and the propensity to develop thrombosis despite anticoagulation, it is decided to change the treatment from heparin to low-molecular-weight heparin (Fragmin 16,000 IU/day) on day 32. After day 55, the patient receives warfarin as prophylaxis against further thromboses and is discharged to her home on day 61. To rule out other causes for the thromboembolic complications, she was tested and found negative for activated protein C resistance.

**Table 1:** Criteria for disseminated intravascular coagulation

	Parameters	Score
Platelet count (10 ^9^ /L)	<50	2
	50–100	1
	>100	0
D-dimer (mg/L)	>2.0	3
	0.2–2.0	2
	<0.2	0
PK (INR)	>1.4	2
	1.2–1.4	1
	<1.2	0
Fibrinogen (g/L)	<1.0	1
	≥1.0	0
Fibrinogen/CRP ratio a	NA	NA
	NA	NA

Abbreviations: CRP, C-reactive protein; NA, not available.

Note: Disseminated intravascular coagulation criteria are fulfilled when the score is ≥ 5 points.

a International Society of Thrombosis and Haemostasis as summarized in Sundberg et al. 8

## Discussion

We hereby wish to highlight the potential risk for thrombosis during HFRS and that the platelet transfusion may have pathogenetically contributed to the event.

HFRS is a mild viral hemorrhagic fever, indicating an increased propensity for bleeding during disease, and nearly one-third of HFRS patients showed mild bleeding manifestations. 9 However, coagulopathy is prominent in HFRS patients with approximately 25% fulfilling overt DIC criteria, 8 increased platelet activation during HFRS, 10 deregulated fibrinolysis, 11 and the identification of HFRS as a risk factor for arterial and venous thrombosis. 5 6 Furthermore, autopsies of patients with fatal HFRS showed thromboses in pulmonary vessels. 12 Other infections have also been shown to carry a similar risk for thromboembolism. 13 14

Platelet activation markers were higher in HFRS patients with thrombosis compared with those who did not have a thrombosis. 10 Furthermore, thrombocytopenia during HFRS is not caused by decreased thrombopoiesis 10 15 but presumably by platelet consumption potentially by adhesion to activated/infected endothelial cells. 16 17 In addition, Puumala virus–infected endothelial cells themselves have a procoagulatory phenotype with increased tissue factor expression. 17 Though this patient in the current case report had severe bleeding, that did not occur during the thrombocytopenic phase but probably secondary to thrombosis and surgery. This case illustrates the coagulopathy and also the propensity for thrombosis in HFRS (as shown by the SMV and PV thrombosis, deep vein thrombosis, and pulmonary embolism) despite being on anticoagulant treatment. However, at the time of treatment of this patient, the increased thrombosis risk was not known and the main focus of treatment was on the predisposition for bleeding.

The risk–benefit of prophylactic platelet transfusion is under debate. 1 In several studies of prophylactic platelet transfusion in Dengue patients with thrombocytopenia, there was no benefit in platelet count recovery or risk of bleeding. 3 4 Instead, the length of hospitalization was increased in patients who received prophylactic platelet transfusion. 3 Furthermore, erythrocyte and platelet transfusion were associated with an increased risk for arterial and/or venous thrombosis in cancer patients, ICU patients, and patients with platelet consumptive and destructive disorders. 18 19 20 In addition, a recent review highlighted that the most common cause of thrombocytopenia in ICU patients was likely platelet consumption, and in such cases transfusing platelets may not be beneficial, possibly even to the point of being harmful. 1 According to this review, the platelet count threshold that was widely accepted for platelet transfusion was 10 × 10 ^9^ /L in patients without additional risk factors such as DIC or severe hepatic or renal dysfunction. If the patient had these risk factors, the platelet count threshold was 20 to 30 × 10 ^9^ /L. 1 Another recent study suggested platelet transfusion in DIC patients with platelet counts less than 20 to 30 × 10 ^9^ /L and at high risk of bleeding. 2 Our patient had a platelet count of 12 × 10 ^9^ /L, DIC, and was suffering from HFRS which are all risk factors for bleeding. Despite this, in our patient bleeding was due to congestion of the ileum, secondary to the splanchnic venous thrombosis.

With this case report, we wish to highlight the difficulty in treating patients with HFRS with the concomitant risk for bleeding and thrombosis. We suggest prophylactic platelet transfusion in patient with HFRS should be kept to a minimum unless the patient shows signs of bleeding.
